# Post-menopausal breast cancer: from estrogen to androgen receptor

**DOI:** 10.18632/oncotarget.22156

**Published:** 2017-10-27

**Authors:** Avisek Majumder, Mahavir Singh, Suresh C. Tyagi

**Affiliations:** ^1^ Department of Biochemistry and Molecular Genetics, University of Louisville School of Medicine, Louisville 40202, Kentucky, USA; ^2^ Department of Physiology, University of Louisville School of Medicine, Louisville 40202, Kentucky, USA

**Keywords:** breast cancer, androgen receptor, estrogen receptor, metastasis, post-menopausal women

## Abstract

In the United States, breast cancer is the second leading cause of death among women, and even though different therapies can treat primary breast tumors, most breast cancer-related deaths (>95%) occur due to metastasis. A majority (~70%) of breast tumors are found to express estrogen receptor, and a significant portion (~90%) of ER-positive (ER^+^) breast tumors are also androgen receptor-positive (AR^+^). Although ER is known to promote tumorigenesis, the role and underlying mechanism(s) of AR in these closely knit processes remain controversial. Endocrine therapies are the most commonly used treatment for patients with ER^+^ breast tumors; but, ~30%-50% of initially responsive patients develop resistance to these therapies. Whereas 70%–90% of all breast tumors are AR^+^ and AR overexpression is correlated with endocrine resistance, but the precise molecular mechanism(s) for this association is yet to be studied. Multiple mechanisms have been proposed to show AR and ER interactions, which indicate that AR may preferentially regulate expression of a subset of ER-responsive genes and that may be responsible for breast cancer and its progression in affected patients. On the other hand, most of the ER^+^ breast tumors found in post-menopausal women (~80%); and they have very low 17β-estradiol and high androgen levels, but how these hormonal changes make someone more prone to cancer phenotype has long been a disputed issue. In this study, we have discussed multiple molecular mechanisms that we believe are central to the understanding of the overall contributions of AR in breast cancer and its metastasis in post-menopausal women.

## INTRODUCTION

In the United States, breast cancer is the second leading cause of death among women (40,610 deaths and 252,710 new cancer cases are estimated in 2017) [1]. There are different therapies (surgery, radiotherapy, chemotherapy, endocrine therapies, or combination of these therapies) available which can treat primary tumor; however, most breast cancer-related deaths occur due to distance organ metastasis (mainly lung, brain and liver). Whereas, most epidemiological studies showed inconsistent finding in a correlation between serum androgen levels and breast cancer risk; but whether this association is consistent in post-menopausal women with breast cancers (who have high androgens levels and low 17β-estradiol) is not understood [2–11]. Previous studies have implicated a possible role for AR in breast cancers [12–14], and AR is found to be predominantly expressed in *in-situ*, invasive and metastatic breast cancers (~90% of primary tumors and 75% of metastases) [15–18]; however, the precise molecular mechanism(s) for AR's contribution to breast cancer is largely unknown. Hence, a detailed understanding of the potential role of AR in breast cancer is essential to design and develop a newer class of treatment option for the patients.

A meta-analysis of 19 studies that included 7693 patients showed that 74.8% of ER^+^ patients were concomitantly AR^+^ while 31.8% of ER^-^ patients were AR^+^ [19], which suggests that AR expression is high in ER^+^ tumors compared to ER^-^ tumors. In addition to this Grogg et al. showed that the expression of AR status is highly conserved during breast tumor progression and is expressed in metastasized breast tumors [20]. Although, ER found to regulate the transcription of genes that promote breast cancer cell proliferation, invasion, and survival [12], the role of AR in these processes is not known. Previous studies showed that AR reportedly decreased ER transcriptional activity [14], probably by competing for some of the same ER binding sites [21]. However, a recent ChIP-seq study demonstrated that treatment of MCF-7 breast cancer cells with the AR antagonists enzalutamide or MJC13 (which directly binds to the ligand-binding domain of AR with higher affinity than dihydrotestosterone) decreased the number of ER genomic binding sites by almost 50%, implicating a role for AR in ER genomic binding in response to 17β-estradiol (E_2_) [21]. This study also found that 75% of E_2_-induced AR-binding sites overlapped with ER-binding sites and surprisingly, when MCF-7 cells (breast cancer cell line) were treated with enzalutamide or MJC13, the genomic binding of ER was only inhibited at those overlapping sites [21], which implies that AR may facilitate ER binding at those loci. In addition to this, some ChIP-seq studies found that AR and ER interactions occur via a long-range enhancer and promoter chromatin interaction [22, 23]. These findings indicate that AR may preferentially regulate expression of a subset of ER-responsive genes based on the ERE sequences and that may induce metastasis.

The endocrine therapy is the most commonly used treatment for patients with ER^+^ breast tumors, which either blocks the E_2_ binding (tamoxifen) or stops the E_2_ production (aromatase inhibitors) [24, 25]. However, ~30-50% of initially responsive patients develop resistance to these endocrine treatments [26, 27], but the precise molecular mechanism of this process is currently unknown [28]. A study by Amicis et al. showed that AR overexpression induces tamoxifen (Tam) resistance in MCF-7 breast cancer cells [29], which suggests that AR may play a role in this process. On the other hand, most of the ER^+^ breast cancer found in post-menopausal women and these patients have very less E_2_ levels and high dihydrotestosterone (DHT) levels under treatment of aromatase inhibitors (AIs) [30, 31]. E_2_ can also be produced via peripheral conversion of androgens to E_2_ by CYP19 aromatase [32]. Although AIs like letrozole, exemestane, and anastrozole have been used for the treatment of these patients, the concentrations of androgens are not altered that much in patients [33–35]. Even though previous reports suggested that androgens levels play a significant role in resistance to this therapy (AIs) [36]; again the precise molecular mechanism(s) are yet to be investigated.

A recent study showed that the AR antagonist enzalutamide, which directly binds the ligand binding domain of AR with higher affinity than dihydrotestosterone (DHT), inhibited PT12 patient-derived xenograft (PDX) tumor growth. Enzalutamide also reduced the number of ‘metastatic’ tumors when injected intra-cardially in female NOD-SCID-IL2Rgc^-/-^ mice strain [21]. ER^+^ breast cancer cell lines that grow as xenograft require exogenous E_2_ supplements [37] whereas, post-menopausal breast cancer patients have very low E_2_ in their blood (<18pg/mL) [38]. PDX tumors are more accurate, but they are very hard to establish from ER^+^ tumors [39] because the engraftment rate is very less (2.5%) [39]. Although these *in vivo* studies suggested that AR has a role in breast cancer metastasis, most of the current *in vivo* breast cancer model systems are not clinically relevant and have many limitations which hinder our understanding of the role of AR in breast cancer metastasis. A preclinical study also showed that the ratio of AR and ER is also an independent predictor of disease-free survival (HR = 4.04, 95% CI: 1.68, 9.69; p = 0.002) and disease-specific survival (HR = 2.75, 95% CI: 1.11, 6.86; p = 0.03) [40]. Anti-androgen drugs have been known for the treatment of triple-negative breast tumors, but there are very fewer data available on ER^+^ tumors. Thus, the range of molecular mechanisms of how AR involves in the regulation of ER transcriptome and what role AR plays in metastasis in ER^+^ tumors is completely unknown. In this review, we have highlighted multiple mechanisms on how AR is involved in breast cancer progression and its metastasis in ER^+^ breast tumors in post-menopausal women. We also emphasize the importance of prognostic implications of AR in breast cancers, potential therapeutic molecules, putative challenges, and related methodologies that deal with post-menopausal AR induced breast cancers. Furthermore, this understanding could also help in identifying novel biomarkers for the detection purposes.

### Androgen receptor expression in different subtypes of breast tumors

**Figure 1:**
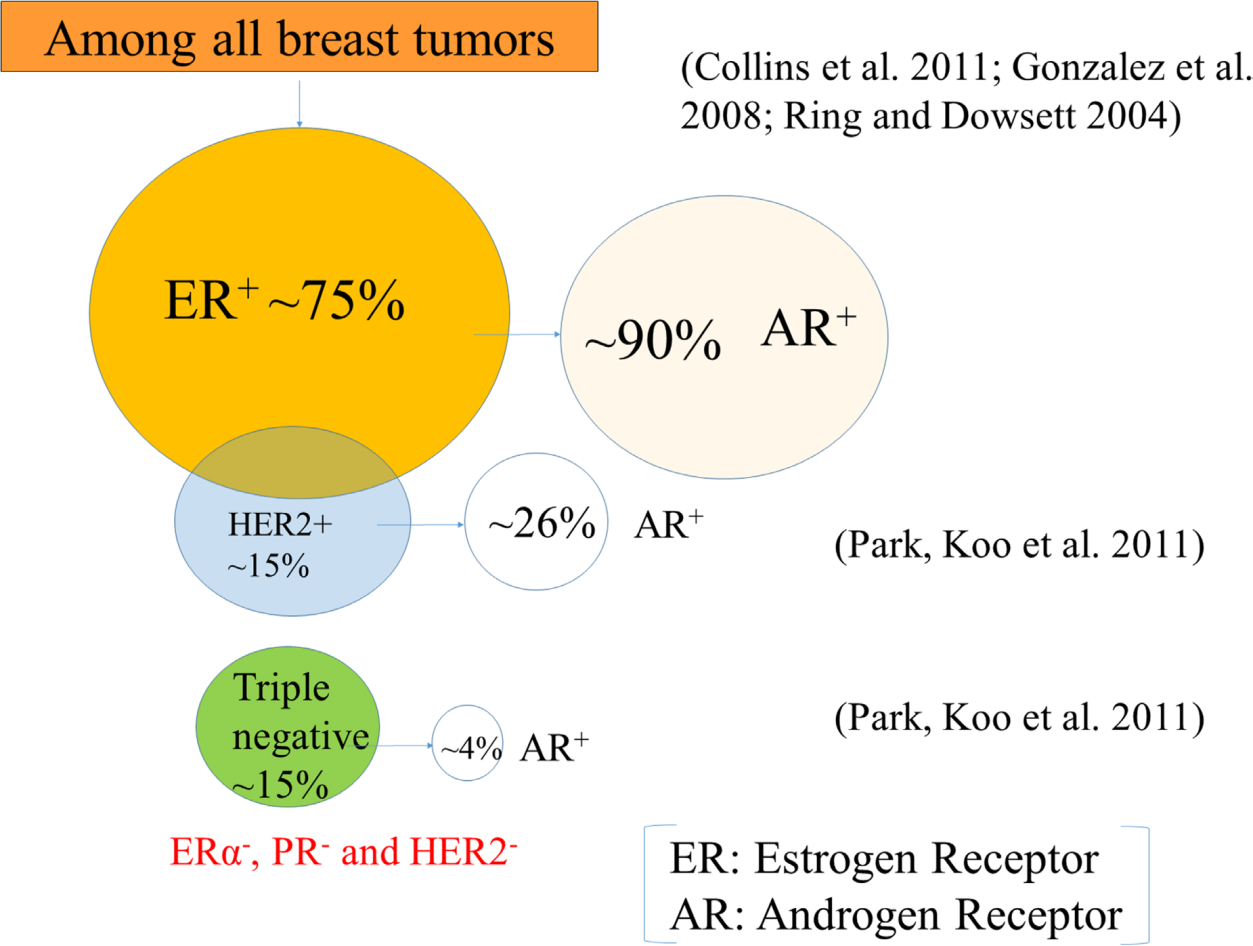
Breast tumor subtypes Roughly 75% of the tumors express the estrogen receptor (ER), and ~15% of the tumors express the human epidermal growth factor receptor 2 (HER2), and almost 15% triple-negative breast cancers do not express ER, progesterone receptor (PR), and HER2. Interestingly, a significant portion (~90%) of ER-positive (ER^+^) breast tumors are also androgen receptor (AR) positive (AR^+^) ones. On the other hand, a very few percentage of HER2+ (26%) and TNBC (4%) express AR.

Breast tumors are mainly classified into two subtypes based on cell types and molecular signatures: (1) luminal breast tumor, which expresses keratin 8/18 (a marker of luminal epithelial cells) and (2) basal breast tumor, which expresses keratin 5/6 (a marker of basal epithelial cells) [41]. However, some of the basal keratin can also be found to express in the luminal cells in the terminal duct lobular units (TDLUs) [42]. Based on gene expression profiling, breast tumors are also classified as luminal A, luminal B, human epidermal growth factor receptor 2 (HER2) and triple negative (Figure 1), and each of them has a different gene expression signature [43]. Luminal A (~40%) expresses ER and/or PR, but doesn't express HER2, and has low levels of a proliferative marker, Ki67 expression [43]. Whereas luminal B (~20%) shows ER and/or PR, and either HER2 positive or HER2 negative and has high levels of a proliferative marker, Ki67 expression [43]. However, HER2 breast tumors don't express ER and PR but express HER2. Breast tumors which express only ER, PR and HER2, are termed as triple-positive, while those that lack their expressions are classified as triple-negative breast cancer (TNBC) [44]. Among all tumors, the majority (~70%) of them express ER [45] and that a significant portion of metastatic tumors also retain their ER status when the primary tumor is ER^+^ [46, 47]. AR expression is lower in ERα^-^ breast tumors and higher in ERα^+^ breast tumors [12, 19].

### Classical and non-classical pathway of AR in breast tumors

**Figure 2:**
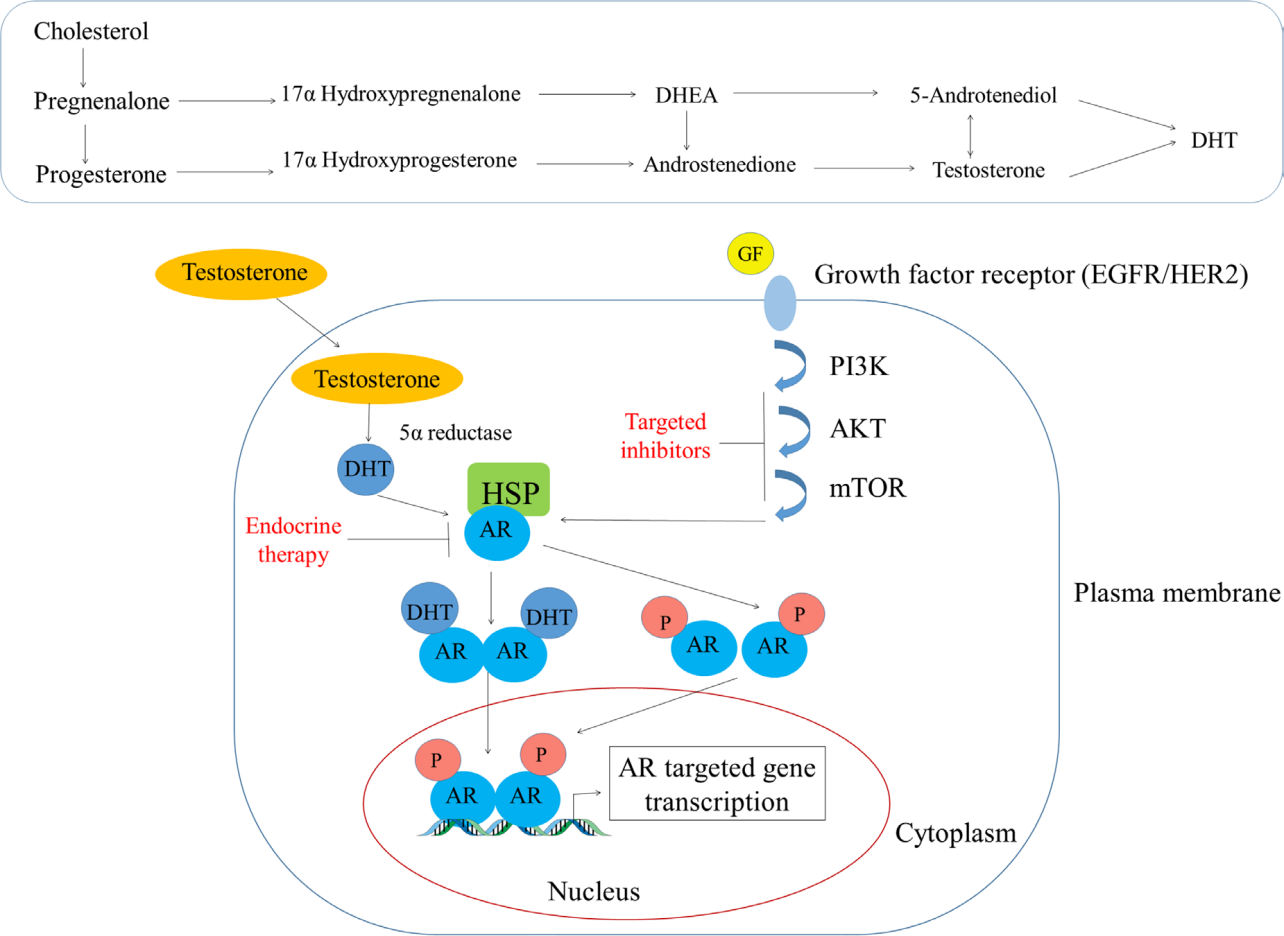
Androgen's mode of action in the breast tissue The top panel shows the conversion of cholesterol to different steroid hormones while the bottom panel depicts how testosterone diffuses through the plasma membrane and interacts with AR that is sequestered in the cytoplasm by the heat shock proteins (HSPs). Subsequently, AR undergoes a conformational change and is released from HSPs and then translocate to the nucleus due to its intrinsic nuclear localization domain. In the nucleus, AR binds to specific DNA motifs (AREs) wherein it recruits co-activators (not shown) to regulate the gene transcription activities.

AR is a type I nuclear receptor which is present in the cytoplasm where it is sequestered by heat shock proteins (HSP-70 and HSP-90) in an inactive state, but when ligands such as androgens bind to its ligand binding domain (LBD) it undergoes a conformational change and is released from the HSPs [48]. Then due to the presence of an intrinsic nuclear localization signal (NLS), AR can translocate to the nucleus where it can bind to specific DNA motifs, and therein it recruits co-activators to regulate transcription of androgen-responsive genes as shown in Figure 2 [48]. In the presence of antagonists, it either recruits co-repressors or prevents co-activators recruitment to AR and represses the transcription of androgen-responsive genes [48]. Testosterone by itself can act as a ligand for AR, or it can also be converted to more potent from DHT via 5α reductase [48]. Testosterone can also get converted to E_2_ by the CYP19 aromatase as shown in Figure 2 [49]. Most of the previous *in vitro* studies on breast cancers have employed DHT (a non-aromatizable form of testosterone) or R1881 (a non-aromatizable synthetic analogue of testosterone) as an agonist for AR, but under normal physiological conditions body has other different androgens like dehydroepiandrosterone (DHEA), androstenedione, androstenediol (A5), androsterone, and DHT; and their binding capacities may different (Table 1). A study by Zava et al. showed that 10 nM concentration of DHT could not induce MCF-7 cell proliferation, whereas they found that 1μM concentration did induce proliferation [50]. Whereas, Bhattacharyya et al. showed that anti-estrogen fulvestrant could effectively suppress AR expression in several human prostate cancer cells (LNCaP, LAPC-4 LN95, and LN97 cells) [51].

**Table 1:** Summary of current literature review on human AR-agonist and antagonist

Agonists/antagonists	Findings	References
**Agonists**: DHT, mibolerone, testosterone, and methyltrienolone	At concentrations between 0.1 and 1 nM induce the AR NH2-terminal and carboxyl-terminal interaction more than 40-fold	Kemppainen, Langley et al. 1999 [1]
**Agonists**: anabolic steroids, oxandrolone and fluoxymesterone	At concentrations between 10-100 nM induce the AR NH2-terminal and carboxyl-terminal interaction for up to 23-fold	Kemppainen, Langley et al. 1999 [1]
**Antagonists**: hydroxyflutamide, cyproterone acetate, RU56187	At concentrations up to 1 μM no interaction detected	Kemppainen, Langley et al. 1999 [1]
**Inhibition androgen synthesis**: ketoconazole and abiraterone	A decline (50%) in prostate-specific antigen was observed in 28 (67%) of 42 phase II patients	Attard, Reid et al. 2009 [2]
**Steroidal antiandrogens**: cyproteroneacetate (CPA), megestrol acetate, andmedroxyprogesteroneacetate	Several drugs toxicity like hepatotoxicity, interference with libido and potency, cardiovascular side effects were observed	Jacobi, Altwein et al. 1980 [3]
**Non-steroidal antiandrogens**: Flutamide	No significant differences in efficacy between Flutamide and CPA monotherapy was detected	Schroder, Whelan et al. 2004 [4]
**Non-steroidal antiandrogens**: Bicalutamide	At a daily oral dose of 25 mg/kg, bicalutamide effected a highly significant reduction in growth of Dunning R3227H transplantable rat prostate tumors	Furr and Tucker 1996 [5]
**Non-steroidal antiandrogens**: Enzalutamide	It showed improvement in overall survival in metastatic castration-resistant prostate cancer post chemotherapy with docetaxel as a first line treatment before initiation of chemotherapy.	Ramadan, Kabbara et al. 2015 [6]

1. Kemppainen JA, Langley E, Wong CI, Bobseine K, Kelce WR, Wilson EM. Distinguishing androgen receptor agonists and antagonists: distinct mechanisms of activation by medroxyprogesterone acetate and dihydrotestosterone. Mol Endocrinol. 1999; 13:440-54. https://doi.org/10.1210/mend.13.3.0255.

2. Attard G, Reid AH, A'Hern R, Parker C, Oommen NB, Folkerd E, Messiou C, Molife LR, Maier G, Thompson E, Olmos D, Sinha R, Lee G, et al. Selective inhibition of CYP17 with abiraterone acetate is highly active in the treatment of castration-resistant prostate cancer. J Clin Oncol. 2009; 27:3742-8. https://doi.org/10.1200/jco.2008.20.0642.

3. Jacobi GH, Altwein JE, Kurth KH, Basting R, Hohenfellner R. Treatment of advanced prostatic cancer with parenteral cyproterone acetate: a phase III randomised trial. Br J Urol. 1980; 52:208-15.

4. Schroder FH, Whelan P, de Reijke TM, Kurth KH, Pavone-Macaluso M, Mattelaer J, van Velthoven RF, Debois M, Collette L. Metastatic prostate cancer treated by flutamide versus cyproterone acetate. Final analysis of the “European Organization for Research and Treatment of Cancer” (EORTC) Protocol 30892. Eur Urol. 2004; 45:457-64. https://doi.org/10.1016/j.eururo.2003.11.016.

5. Furr BJ, Tucker H. The preclinical development of bicalutamide: pharmacodynamics and mechanism of action. Urology. 1996; 47:13-25; discussion 9-32.

6. Ramadan WH, Kabbara WK, Al Basiouni Al Masri HS. Enzalutamide for patients with metastatic castration-resistant prostate cancer. Onco Targets Ther. 2015; 8:871-6. https://doi.org/10.2147/ott.s80488.

AR can also be activated by non-genomic signaling pathways such as PI3kinase, Akt, and m-TOR [52] (Figure 2). Except these, a study showed that AR could also be regulated by ERK-signaling pathway [53, 54]. Targeting this pathway has been demonstrated to impair androgen-stimulated tumor cell growth, suggesting that targeting this signaling pathway could be a potential therapeutic approach to treat ER-/HER2+ breast cancers [55]. Another study showed bicalutamide; an AR antagonist can block the growth of MDA-MB-453 human breast cancer cells by blocking HER2/HER3 signaling and downstream effectors PI3K/AKT [56]. AR can also found to regulate gene expression through mitogen-activated protein kinase (MAPK) signaling pathway in ERα/PR-negative MCF-10A cells [57]. Some studies also reported that AR induces cell proliferation, migration, and apoptosis in prostate and breast cancer through PTEN cell-signaling pathway [58]. In addition to this, a similar study by this group also showed that AKT and PTEN negatively regulate each other via the transcription factor GATA2 [59]. However, more extensive studies need to be done to target specific pathways which could help to treat post-menopausal women with breast cancer.

### The role of AR in the resistance to endocrine therapies

The endocrine therapyis the most commonly used treatment for patients with ER^+^ breast tumors, which either blocks the E_2_ binding (tamoxifen) or stops the E_2_ production (AIs) [24, 25]. However, ~30-50% of initially responsive patients develop resistance to these endocrine treatments [26, 27], but precise molecular mechanisms of this process are currently unknown [28]. Previous studies showed that ER^+^ tumors which respond to neoadjuvant endocrine therapy has less AR mRNA levels, and tumors which don't respond to this treatment have shown a low level of AR mRNA levels [60, 61]. Similarly, another study demonstrated that resistance to Tam correlates with AR overexpression in both *in vivo* and *in vitro* experiment [29]. Since a majority (~70%) of breast tumors found to express ER and a significant portion (~90%) of ERα^+^ breast tumors are AR^+^ [62–66], but it's still unknown whether AR plays any role in this process. A majority of ER^+^ tumors eventually develop into a more aggressive phenotype and in an E_2_ independent phenotype without undergoing an alteration of ER status [67]. Whereas, most of the ER^+^ breast tumors are found in post-menopausal women (~80%), and the normal range of DHT and E_2_ in postmenopausal women is 30-62pM/L and 55-220 pM/L, respectively, but at the same time DHT is increased, and E_2_ is decreased in ERα^+^ breast cancer patients on the AIs anastrozole [30, 31]. Additionally, androgens are found to associate with poor prognosis via inducing EGFR (epidermal growth factor receptor) signaling. In genomic pathway, ER is activated by E_2_, however as previously described androgen can also be converted to E_2_ by aromatase enzyme (CYP19) [33], which makes this interplay between these two hormones even more complicated. Most of the previous studies that have been done so far regarding the role of AR in metastasis of ER^+^ tumors used exogenous E_2_ and DHT stimuli [68]. So, it's very important to determine whether AR promotes metastasis of ER^+^ breast cancer cells in the absence of E_2_ as a model of postmenopausal women with breast cancer (80% of ER^+^ breast tumors patients) treated with an aromatase inhibitor (AIs) [38] or in the presence of both E_2_ and DHT (where ER is fully active). An ER ChIP-seq study found that ERα-binding to chromatin still occurred in ERα^+^ Tam-resistant MCF-7 (MCF7-TamR) cells, implying that TamR was not due to loss of ER binding to DNA [69]. Moreover, genomic binding of ER was found to increase in metastatic tumors compared to primary tumors [69]. An enriched motif analysis showed that ER binding sites in EREs were increased in Tam-R MCF-7 cells and those were the same motifs observed in the ERα^+^ metastatic tumors (collected from breast cancer patients) [69]. As Enzalutamide reduced the E_2-_induced proliferation of MCF7-TamR cells and decreased the growth of MCF7-TamR cells in soft agar [21], suggesting that there is a good opportunity to use AR-antagonist as a therapeutic for patients who don't get a response to Tam. In future, we encourage more study to determine whether by blocking AR can prevent ER-regulated metastasis Tam-R ER^+^ breast cancer patients through a unique molecular mechanism other than anti-estrogen therapies.

### The role of AR in ER^+^ breast tumors of post-menopausal women

Like AR, ER is a type 1 nuclear receptor, and upon its activation, through both genomic and non-genomic mechanisms, it induces transcription of estrogen-responsive genes [70]. There are two subtypes of ER, ERα and ERβ [70]. In healthy breast tissue, ERβ expression is found to be high, whereas during breast cancer ERα expression is increased [71]. In the genomic pathway, E_2_ acts as ERs ligand [70], however as previously described androgen can also be converted to E_2_ [33] (Figure 2) and can serve as an ER ligand. In a study, Szelei et al. showed that higher levels of AR expression are correlated with less proliferation of MCF-7 cell line [72], which suggests that AR may help in the progression of ER^+^ tumors. Similarly, another study showed that androgens induce apoptotic activity and also down-regulate proto-oncogenes bcl-2 in MCF-7, ZR75-1 and T47-D cells [8, 73]. A ChIP study demonstrated that ERα assembled on the AR gene promoter and this was associated with elevated basal expression of AR mRNA through EGFR/MAPK pathway in Tam-R MCF-7 cells [74]. Similarly, Kumar et al. showed that co-expression of ER and AR reduced the AR transcriptional activity by increasing concentrations of ER cDNA in the presence of both of DHT and E_2_ in QT6 cells [75]. AR can also bind to EREs, and down-regulate ERα transcriptional activity by competing for the same binding EREs [13]. A ChIP-seq and gene microarray analysis of the ZR-75-1 luminal breast cancer cell line proved that in the presence of higher concentration of one ligand (DHT versus estradiol) over the other could lead to either an increase or decrease in the ER/AR transcriptional activity [76]. For example, if AR binds to EREs it leads to an anti-proliferative effect rather than the proliferative effect of ERα binding to ERE and vice-versa for ERα binding to androgen response elements (AREs). Suzanne et al. showed that AR contributed to ERα transcriptional activity in ER^+^ MCF-7 cells by overexpressing aromatase (CYP19) and AR [77]. Another study by Richer et al. demonstrated that AR inhibitor Enzalutamide inhibited DHT- driven MCF-7 cells proliferation *in vitro* as well as tumor growth in both ER^+^ (MCF7) and ER- (MDA-MB-453) xenografts [40].

### AR and ER cross-talk in breast cancers of post-menopausal women

**Figure 3:**
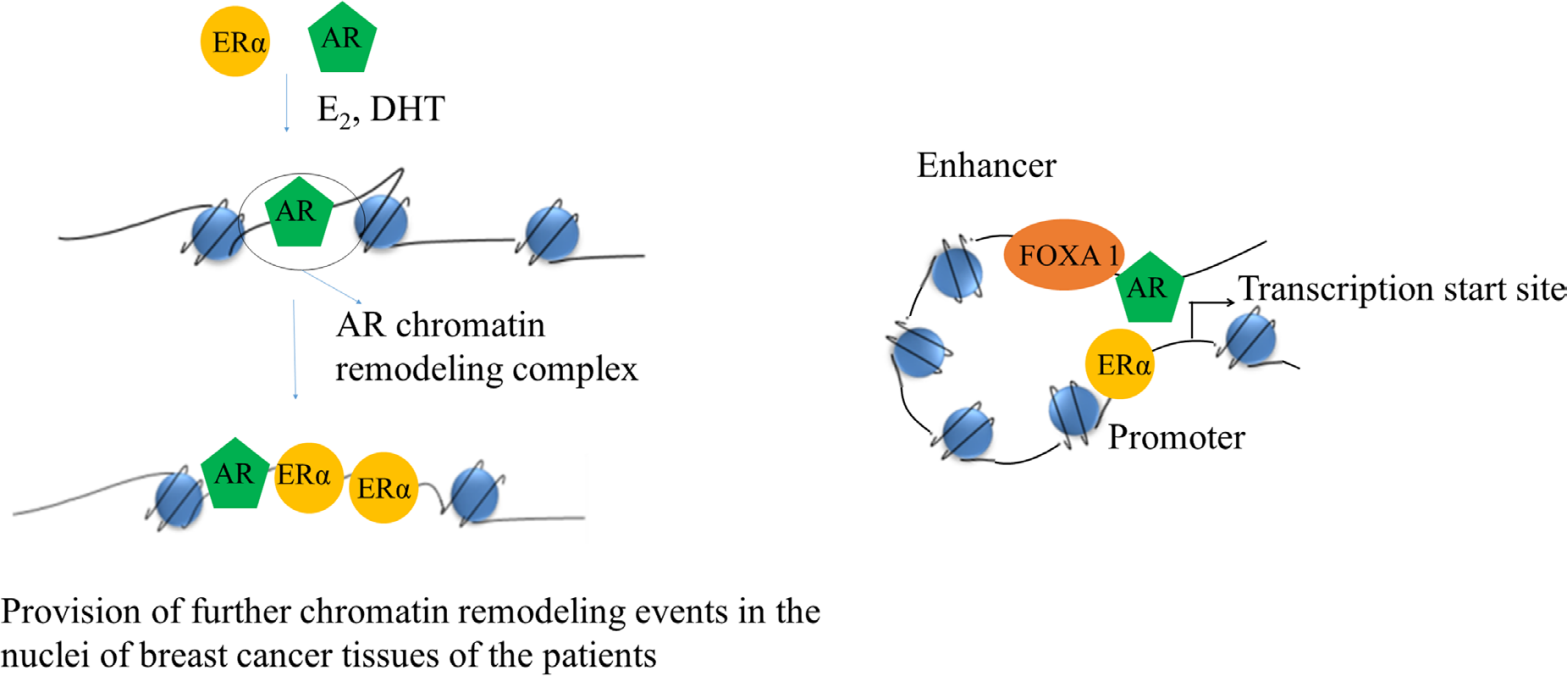
Schematic of a cartoon depicting AR and ERα interaction On the left its showing how AR can increase accessibility of ERα to the proximal regulatory regions of ERα responsive genes. On the right side, it's showing how AR may also interact with ERα via long-range chromatin interaction.

Previous studies showed that ER and AR co-localized in ER^+^ malignant human breast epithelial cells [13], suggesting that they may interact in the same cell. Metastasis is a multistep process and requires cell proliferation, motility, invasion, and survival of breast cancer cells [78, 79]. Although ERα has been found to regulate the transcription of genes that promote breast cancer cell proliferation, motility, invasion, and survival [12], the role AR in these processes is controversial [13, 14, 57, 75, 80, 81], and may depend on the hormonal milieu. Although targeting of AR has been used in prostate cancer therapy, very less information is available on the therapy against breast cancer and its prognosis. Some recent studies did show that AR can modulate the genomic signaling of ER in MCF-7 cells [82]. Another study demonstrated that co-expression of AR with ERα decreased ERα transactivation by 74% and they also showed that amino-terminal domain of AR interacts with ligand-binding domain of the ERα [14]. Previous studies reported that AR could also bind to EREs, and shown to downregulate ERα transcriptional activity by competing for binding to the same EREs sequences [21]. However, a recent ChIP-seq study of ER binding sites demonstrated that treatment of MCF-7 breast cancer cells with the AR antagonist enzalutamide decreased the number of E_2_-induced ER genomic binding sites by 50%, implicating a role for AR in ER genomic binding [21]. This study also showed that 75% of the estradiol (E_2_)-induced AR-binding sites overlapped with ER-binding sites and enzalutamide inhibited ER binding only at those overlapping sites [21], which suggests that AR may facilitate genomic binding of ER at those particular genomic loci. AR binds to the consensus EREs *in vitro* and is recruited to the promoters of E_2_-regulated genes containing non-consensus EREs with DHT plus E_2_ treatment in T47D cells; however, AR-ER doesn't form a heterodimer complex [13]. Hence, one of the possible mechanisms may be that AR may induce some pioneer factor to increase the accessibility ER to the proximal regulatory regions of ER-responsive genes as shown in Figure 3. In addition to this, some ChIP-seq studies found that AR and ER interactions occur via a long-range enhancer along with promoter chromatin interaction [22, 23]. A study also showed that forkhead box (FOX) family member (FOXA1) binding in a subset of genes are the same as that of AR binding sites [83] and silencing of FoxA1 inhibits AR binding in MDA-MB-453 cells [84], which suggests that FOXA1 may act as a pioneer factor for AR. Some studies showed that FOXA1 binding helps H3K4 mono-methylation at enhancers, suggesting that AR may induce ER genomic binding via a long-range chromatin interaction as illustrated in Figure 3 [85–87]. These studies indicate that AR may regulate genomic binding of ER through recruiting some pioneer factors like FOXA1 and regulate transcription of a subset of genes, which may induce breast cancer in post-menopausal women.

**Figure 4:**
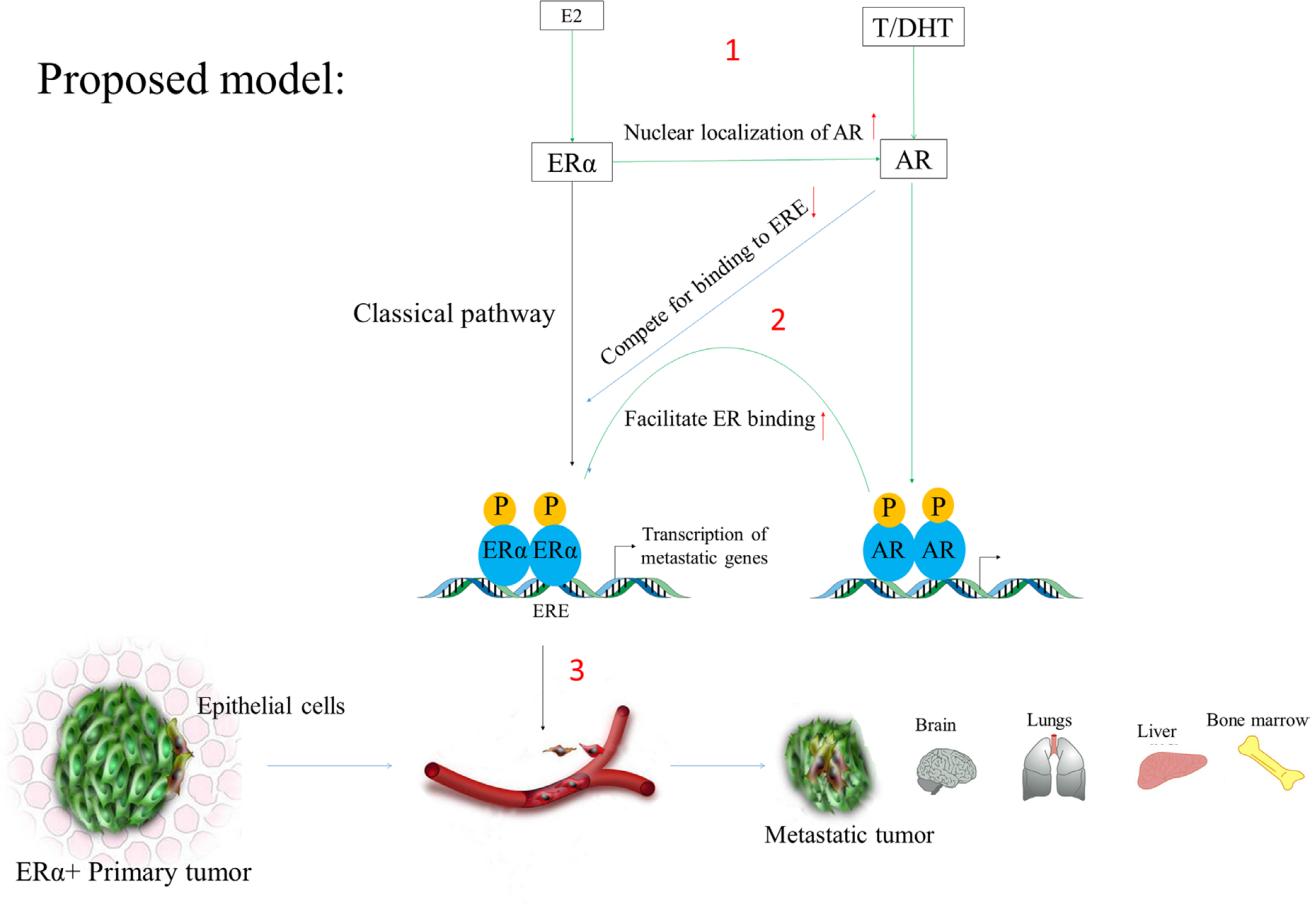
The classical pathway showing E2 and DHT action In this scheme, AR induces metastasis by regulating ERα transcriptome via recruiting ERα to the promoters of the genes responsible for the metastatic phenotypes. (1) The left side indicates that when E_2_ binds to ERα it (ERα) it gets recruited to the promoter of ER-responsive genes (EREs). Similarly, DHT stimulates AR activation and its recruitment to the specific DNA motifs. (2) Since AR can also binds and compete to EREs and as well as it can facilitate ER binding via recruitment of other pioneer factors like FOXA1 or long-range promoter-enhancer interaction (need to be further research). (3) In this way, the associated gene expression changes may induce breast cancer phenotypes and its subsequent metastasis in post-menopausal women with breast cancer patients.

Previous studies have already been reported that ER promotes transcription of genes which cause breast cancer and its metastasis [12], and the AR inhibitor Enzalutamide reduced ERα genomic binding by 50% [21], but how AR regulates ER transcriptome in ER^+^ breast cancer cells and whether it induces metastasis is not known. So it's essential to further study by a combined approach of microarray, RNA-seq, ChIP-seq, and ATAC-seq to determine which sets of genes regulated by AR in ER^+^ breast tumors. There are many potential genes which can be directly regulated by AR as shown in Figure 4, and which can induce breast cancer and its metastasis as listed in Table 2 (more genes can be found in MetaCore and ERGDB database). However, AR can also indirectly regulate ER-responsive genes. For example, HMG20B (transcribed from AR-responsive genes) can form a heterodimer with HMG20A, whereas HMG20A found to stabilize the ERα binding and has a role in EMT [88]. So these genes need to be checked by RNA-seq analysis between AR^+^ and AR knockout ER^+^ breast cancer cell line. Previously studies showed that in the presence of DHT membrane bound-AR activates AKT signaling, which subsequently activated ERα [89]. Therefore, AKT-phosphorylation status after treatment with AR ligands also needs to be checked. We also encourage further research to identify novel genes and pathways, which were not previously reported to induce breast cancer; and AR-regulated lincRNA, lncRNA and miRNAs (miRTarBase web tool) which may cause breast cancer in post-menopausal women.

**Table 2:** List of genes which are involved in breast cancer and its metastasis

Study type	Methods	Gene name-fold change (p value)	References
Highly modulated transcripts in ERα^+^ breast carcinomas	This *in vivo* experiment was performed on 26 Stage I – Stage II invasive breast carcinomas (8 ERα^-^ tumors and 18 ERα^+^). Serial Analysis of Gene Expression based on ERα status is reported.	**Cell proliferation related:** TFF1 (trefoil factor 1) - 51.4 (0.0016) DUSP4 (dual specificity phosphatase 4)- 14.7 (0.0016) NDN (necdin homolog)- 13.3 (0.0026) HDGFRP3 (hepatoma-derived growth factor)- 9.8 (0.0019) TSPAN1 (tetraspan 1)- 9.5 (0.0017) SEP6 (septin 6)- 7.6 (0.0044) DHX34 (DEAH box polypeptide 34)- 7.1 (0.0129) **Apoptosis related:** CARD10^*^ (caspase recruitment domain family)- 11.1 (0.0030) **Signal transduction related:** SYTL4^*^ (synaptotagmin-like 4)- 28.0 (0.0003) ECM1^*^ (extracellular matrix protein 1)- 10.1 (0.0175) LEPR^*^ (leptin receptor)- 9.8 (0.0302) PTGES (prostaglandin E synthase)- 8.0 (0.0168) SCUBE2 (signal peptide, CUB domain EGF-like 2)- 7.5 (0.0024) ADORA2A^*^ (adenosine A2a receptor)- 7.1 (0.0460) ITGBL1 (integrin beta-like 1)- 7.1 (0.0159) **Regulation of transcription related:** ESR1 (estrogen receptor 1)- 9.8 (0.0000) TCEAL1 (transcriptional elongation factor A)- 9.8 (0.0014) ZNF14 (zinc finger protein 14)- 8.4 (0.0023) ZNF38^*^ (zinc finger protein 38)- 7.6 (0.0051) HIF1AN^*^ (hypoxia-inducible factor 1α subunit inhibitor)- 7.1 (0.0094) HOXC13 (homeo box C13)- 7.1 (0.0157) **Cytoskeleton:** MAPT (microtubule-associated protein tau)- 9.8 (0.0085) MYLIP (myosin regulatory light chain interacting)- 9.3 (0.0036) **Metabolism and miscelaneous:** RIMS4 (regulating synaptic membrane exocytosis)- 24.9 (0.0378) NAT1 (N-acetyltransferase 1)- 11.7 (0.0385) ATP6V1B1^*^ (ATPase, H+ transporting)- 10.7 (0.0111) JDP1 (J domain containing protein 1)- 10.0 (0.0035) CHST11 (carbohydrate sulfotransferase 11)- 9.8 (0.0009) CILP (nucleotide pyrophosphohydrolase)- 9.3 (0.0054) ABCA3 (ATP-binding cassette sub-family A)- 8.9 (0.0149) SEC14L2- 8.7 (0.0487) ANXA9^*^ (annexin A9)- 8.4 (0.0145) KCTD3 (K channel tetramerisation domain 3)- 8.4 (0.0001) SFRS7 (splicing factor)- 8.0 (0.0031) SNRPA^*^ (small nuclear ribonucleoprot. polypep. A)- 7.6 (0.0009) NNMT (nicotinamide N-methyltransferase)- 7.6 (0.0120) SLC1A4 (solute carrier family 1 member 4)- 7.6 (0.0254) TIPARP (TCDD-inducible polymerase)- 7.6 (0.0051) SLC7A2 (solute carrier family 7 member 2)- 7.3 (0.0190) GA^*^ (liver mitochondrial glutaminase)- 7.1 (0.0126) **Unknown function:** C1orf34- 13.3 (0.0025) SMILE (hypothetical protein FLJ90492)- 11.1 (0.0004) RHBDL4 (rhomboid, veinlet-like 4)- 10.7 (0.0099) KIAA0882-10.1 (0.0007) C20orf103-9.3 (0.0277) FLJ33387- 9.3 (0.0118) TRALPUSH- 8.9 (0.0458) KIAA0980- 7.6 (0.0096) C10orf32-7.3 (0.0002) FLJ13611-7.1 (0.0069)	Abba, Hu et al. 2005 [1]
Genes altered in co-cultured cancer cells in response to estrogen; Fold change was consider on treatment with E_2_ (10^−8^ M) over untreated (EtOH)	This *in vitro* study was performed on MDA-MB-231 (ER-negative human breast cancer cell line) cells stably transfected with ERα or ERβ. They used array-based expression profiling and quantitative RT-PCR to study the gene expression profiles.	**Fold changes in MDA-MB-231 cells stably transfected with ERα:** Macrophage myristoylated alanine-rich C kinase substrate (MacMarcks)- 7.3 Fibroblast growth factor 1 (FGF1) 3.8 Tissue inhibitor of metalloproteinase 3 (TIMP3) 2.9 Osteopontin (SPP1) 2.8 Cyclin D1 (CCND1) 2.2 Aromatase p450 (CYP19) 2.0 Secretoglobin 2A1 (SCGB2A1) 0.5 Secretoglobin 2A2 (SCGB2A2) 0.5 Integrin alpha 3 (ITGA3) 0.5 Ehm2 (EHM2) 0.4PEA3 (ETV4) 0.4 Urokinase plasminogen activator (uPA) 0.4 Matrix metalloproteinase 2 (MMP2) 0.4 Plasminogen activator inhibitor 2 (PAI2) 0.4 Fibroblast growth factor 2 (FGF2) 0.3 Intercellular adhesion molecule 5 (ICAM5) 0.3 Neural cell adhesion molecule 1 (NCAM1) 0.3 **Fold changes in MDA-MB-231 cells stably transfected with ERβ:** Deleted in colorectal carcinoma (DCC) 0.2 Vascular endothelial growth factor C (VEGFC) 0.5 Kangai 1 (KAI1) 0.5 Matrix metalloproteinase 9 (MMP9) 0.5 Transforming growth factor alpha (TGFA) 0.5 Intercellular adhesion molecule 5 (ICAM5) 0.5 Vascular endothelial growth factor (VEGF) 0.5 Fibroblast growth factor 1 (FGF1) 0.5 Matrix metalloproteinase 8 (MMP8) 0.4c-fes (FES) 0.4 Mucin 1 (MUC1) 0.4 Matrix metalloproteinase 2 (MMP2) 0.4 Neural cell adhesion molecule 1 (NCAM1) 0.3 Vitronectin (VTN) 0.3 Transforming growth factor beta 1 (TGFB1) 0.3 Thrombospondin 2 (THBS2) 0.1	Wang, Jarrett et al. 2007 [2]
Genes involved in breast cancer metastatic to bone	This *in vivo* study was performed on 107 primary breast tumors in patients. They used microarrays based approach to study the gene expression profile.	Trefoil factor 1 (TFF1) 3.1 Trefoil factor 3 (TFF3) 2.6 Anterior gradient 2 homolog (AGR2) 1.9 N-Acetyltransferase 1 (NAT1) 2.5 Cysteine-rich protein 1 (CRIP1) 1.9 Trinucleotide repeat containing 9 (TNRC9) 1.9 Pp14571 2.0 Signal peptide, CUB domain, EGF-like 2 (SCUBE2) 2.1 Trinucleotide repeat containing 9 (TNRC9) 1.9 Cytochrome P450, family 2, subfamily B, polypeptide 6 (CYP2B6) 2.1 Rho family GTPase 1 (RND1) 1.7dynein, axonemal, light Intermediate polypeptide 1 (DNALI1) 2.0 Kinesin family member 5C (KIF5C) 2.0 Trinucleotide repeat containing 9 (TNRC9)- 1.9 Phospholipase A2, group IVB (PLA2G4B)-1.8 Uracil-DNA glycosylase 2 (UNG2)- 1.73-Hydroxy-3-methylglutaryl-coenzyme A synthase 2 (HMGCS2)-2.3 Solute carrier family 1 member 1 (SLC1A1)-2.7 Carcinoembryonic antigen-related cell adhesion molecule 6 (CEACAM6) 2.3 Tetraspan 1 (TSPAN-1) 1.8 RALBP1 associated Eps domain containing 2 (REPS2)- 1.8 Hemopexin (HPX) 1.7 Phosphodiesterase 4D interacting protein (PDE4DIP) 2.2 Carcinoembryonic antigen-related cell adhesion molecule 6 (CEACAM6) 2.8 Target of myb1-like 1 (TOM1L1)- 1.8 Secretoglobin, family 2A, member 2 (SCGB2A2)-1.9 Annexin A9 (ANXA9)- 1.7 Breast carcinoma amplified sequence 1 (BCAS1)-1.8 Tissue inhibitor of metalloproteinase 4 (TIMP4)-1.8 Cytochrome P450, family 2, subfamily B, polypeptide 6 (CYP2B6)-1.8 Chromosome 6 open reading frame 29 (C6orf29) 1.7 Chromosome 9 open reading frame 116 (C9orf116) -1.9 Microseminoprotein beta (MSMB) -3.0 Dual specificity phosphatase 4 (DUSP4) -1.9 Fibroblast growth factor receptor 3 (FGFR3) -1.8 Fibroblast growth factor binding protein 1 (FGFBP1) -1.9 BCL2-associated athanogene 2 (BAG2)- 1.8 Forkhead box O3A (FOXO3A)-2.6 Engrailed homolog (EN1)-2.5 Keratin 16 (KRT16)-12.7 BENE protein (BENE)- 1.9 Potassium voltage-gated channel, subfamily G, member 1 (KCNG1)- 3.4 Immunoglobulin lambda constant2///Immunoglobulin lambda joining 3 IGLC2///IGLJ3)- 2.7 Kallikrein 8 (KLK8)-1.8 Immunoglobulin heavy chain V region(Humha448)///similar to Ig heavy-chain V-I region HG3 precursor 2.4 Keratin 6B (KRT6B)- 2.5 Snail homolog 1 (SNAI1)- 1.8 Thymosin, beta, identified in neuroblastoma cells (TMSNB)- 2.3 Neurturin (NRTN)-2.7 EPH receptor B3 (EPHB3)-2.0 Enolase 1 (ENO1)- 2.3 Superoxide dismutase 2, mitochondrial (SOD2)-1.9 Ig heavy constant gamma 1 (IGHG1)- 1.9 Kallikrein 5 (KLK5)-2.2 Retinoic acid receptor responder 1 (RARRES1) -2.2 Tweety homolog 1 (TTYH1) 5.0 CD24 antigen (CD24)- 2.1 MGC27165///IGHG1 (Hypothetical protein MGC27165///immunoglobulinheavy constant gamma 1)- 2.1 SERPINB5 (Serine (or cysteine) proteinase inhibitor, clade Bmember 5)- 2.5 Ubiquitin carboxyl-terminal esterase L1 (UCHL1) – 2.6 Ropporin, rhophilin-associated protein 1 (ROPN1)-2.6 NADH:ubiquinone oxidoreductase MLRQ subunit Homolog (LOC56901)- 2.4 Integrin, alpha 6 (ITGA6)- 1.9ubulin, beta polypeptide (TUBB///MGC8685)- 2.1 Ubiquitination factor E4B (UBE4B)-1.9 Immunoglobulin heavy chain VH3 (IGHM)-2.0 Collagen, type II, alpha 1 (COL2A1)- 4.4 Inositol(myo)_1(or 4)-monophosphatase 2 (IMPA2)-1.9 DRE1 protein (DRE1)-1.8 Kallikrein 7 (KLK7)-4.0 TTranscriptional coactivator with PDZ-binding motif (TAZ)- 2.0 Ubiquitin specific protease 34 (USP34)- 1.8 Chromosome 6 open reading frame 4 (C6ORF4) Aminolevulinate, delta-, dehydratase (ALAD) Rho GDP dissociation inhibitor alpha (ARHGDIA) Arsenate resistance protein (CLCA2) Calmegin (CLGN) TTranscriptional coactivator with PDZ-binding motif (TAZ)- 2.0 Ubiquitin specific protease 34 (USP34)- 1.8 Chromosome 6 open reading frame 4 (C6ORF4) Aminolevulinate, delta-, dehydratase (ALAD) Rho GDP dissociation inhibitor alpha (ARHGDIA) Arsenate resistance protein (CLCA2) Calmegin (CLGN) Engulfment and cell motility 2 (ELMO2) Leucine rich repeat containing 31 (LRRC31) HIV TAT-specific factor 1 (HTATSF1) 5-Hydroxytryptamine (serotonin) receptor 2B (HTR2B) Melanophilin (MLPH) Solute carrier family 2, member 8 (SLC2A8) Tissue factor pathway inhibitor 2 (TFPI2)	Smid, Wang et al. 2006 [3]
Metastasis-associated protein 1 (MTA1) MTA1 overexpression correlates significantly with tumor grade and angiogenesis in human breast cancers.	This *in vivo* experiment was done on 263 surgical specimens of breast cancer cases and IHC was performed to know the MTA1 expression level.	MTA1 overexpression correlated significantly with higher tumor grade (grades 1 and 2 vs grade 3, P = 0.009).	Jang, Paik et al. 2006 [4]
Reduced expression of the breast cancer metastasis suppressor 1 (BRMS1) mRNA is correlated with poor progress in Breast Cancer.	This *in vivo* experiment was performed on 161cases of invasive carcinoma of the breast and BRMS1 mRNA was assessed by Light Cycler quantitative real-time reverse transcription-PCR.	They found that higher BRMS1 expression was correlated with better prognosis and overall disease-free survival in breast cancer.	Zhang, Yamashita et al. 2006 [5]
Genes differentially expressed between primary breast cancer and breast cancer brain metastasis	This *in vivo* experiment was performed on 44 patients with brain metastasis from breast cancer and expression was measured on the NanoString nCounter Analysis System (NanoString Technologies, Seattle, WA, USA).	MME (Membrane Metalloendopeptidase) MMP2 (Matrix Metallopeptidase 2) CXCL12 (C-X-C motif chemokine ligand 12) PDGFRA (Platelet Derived Growth Factor Receptor Alpha) VCAM1 (Vascular cell adhesion protein 1) MMP13 (matrix metallopeptidase 13) ITK (IL2 inducible T cell kinase) MMP11 (matrix metallopeptidase 11) MMP9 (matrix metallopeptidase 11) SPIB (Spi-B transcription factor) SCUBE2 (signal peptide, CUB domain and EGF like domain containing 2) TP63 (tumor protein p63) FLT3 (fms related tyrosine kinase 3) JAK3 (Janus kinase 3) KRT14 (keratin 14) KRT17 (keratin 17) KRT5 (keratin 5) SFRP1 (secreted frizzled related protein 1) SPIC (Spi-C transcription facto) PGR(progesterone receptor)	Lee, Park et al. 2016 [6]

1. Abba MC, Hu Y, Sun H, Drake JA, Gaddis S, Baggerly K, Sahin A, Aldaz CM. Gene expression signature of estrogen receptor alpha status in breast cancer. BMC Genomics. 2005; 6:37. https://doi.org/10.1186/1471-2164-6-37.

2. Wang J, Jarrett J, Huang CC, Satcher RL Jr, Levenson AS. Identification of estrogen-responsive genes involved in breast cancer metastases to the bone. Clin Exp Metastasis. 2007; 24:411-22. https://doi.org/10.1007/s10585-007-9078-6.

3. Smid M, Wang Y, Klijn JG, Sieuwerts AM, Zhang Y, Atkins D, Martens JW, Foekens JA. Genes associated with breast cancer metastatic to bone. J Clin Oncol. 2006; 24:2261-7. https://doi.org/10.1200/jco.2005.03.8802.

4. Jang KS, Paik SS, Chung H, Oh YH, Kong G. MTA1 overexpression correlates significantly with tumor grade and angiogenesis in human breast cancers. Cancer Sci. 2006; 97:374-9. https://doi.org/10.1111/j.1349-7006.2006.00186.x.

5. Zhang Z, Yamashita H, Toyama T, Yamamoto Y, Kawasoe T, Iwase H. Reduced expression of the breast cancer metastasis suppressor 1 mRNA is correlated with poor progress in breast cancer. Clin Cancer Res. 2006; 12:6410-4. https://doi.org/10.1158/1078-0432.ccr-06-1347.

6. Lee JY, Park K, Lee E, Ahn T, Jung HH, Lim SH, Hong M, Do IG, Cho EY, Kim DH, Kim JY, Ahn JS, Im YH, et al. Gene Expression Profiling of Breast Cancer Brain Metastasis. Sci Rep. 2016; 6:28623. https://doi.org/10.1038/srep28623.

### Androgen to estrogen dependence in breast cancers of post-menopausal women

It has been known that androgenic hormones (mainly testosterone, dihydrotestosterone and androstenedione) are the major circulating sex hormones in females, which are directly or metabolized into a different form and thereby acting as AR ligands. After menopause, the E_2_ level goes down to 10 folds, whereas androgens levels decrease only 1.5-fold [90]. Although these excess levels of androgens may reflect increased availability of androgenic ligands [91], breast tissue can metabolize androgens to E_2_ by enzyme CYP19 aromatase, which is one of the most potent ligands of ER [92, 93]. A study showed that when male mice treated with aromatase inhibitor, AR expression was increased compared to the control mice [94], suggesting that post-menopausal women may express more AR than pre-menopausal women or it could be that women after menopause express more aromatase for the production of E_2_, which may lead to breast cancers [95]. Similarly, previous studies showed that post-menopausal breast cancer patients have high testosterone levels and high expression of AR, which were significantly associated with the ER^+^ status of the tumors (OR 2.42; 95% CI, 1.22-4.82) [10, 11]. Although previous studies showed that patients with AR^+^ breast tumors showed prolonged survival and better response to hormone treatments, as androgens levels are positively associated with breast cancer risk in post-menopausal women, suggesting that It may be the AR expression that correlates with ER expression, but more studies are needed to prove this. Molecular apocrine tumors were known to express AR but not ER, however studies showed that these tumors have an expression profile like that of ER^+^ luminal breast tumors, and the likely mechanism could be through the AR signaling pathway [84]. Therefore, similar mechanisms may be responsible when ER^+^ breast cancer patients become resistant to endocrine therapies.

### AR as a therapeutic target for breast cancer in post-menopausal women

Breast cancer patients have been treated with targeted therapies or endocrine therapies; however, many patients are not eligible for these treatments because their tumors lack expression of these receptors, and/or their tumors have become resistant to these therapeutic agents. In the first half of 20^th^ century, androgen therapy has been used for breast cancer treatment in post-menopausal women; however, this treatment has been discontinued after some studies showed that androgen could be converted to estrogen [96]. A recent clinical study showed that among 116 cases of invasive breast cancer AR was expressed in 56% of the cases and AR expression was significantly associated with early stage (p < 0.03) of breast cancer [8]. Other clinical studies also showed AR expression is higher (n = 573, 85%) among all breast cancer, however, multivariable analysis for short-term follow-up indicated higher metastasis among patients with AR+ER- tumors (HR 3.5; 95% CI 1.4-9.1) than other (AR and ER combinations).

A previous study has demonstrated that androgen therapy was slightly less effective than estrogen-based therapy [97]. However, a few clinical trials have been conducted for the AR-directed therapy in breast cancers [98–100] and information of all drugs which were used in clinical trials are described in Table 3. Additionally, with the availability of more data regarding the presence of AR and the mechanism of its action regarding androgens in breast cancers, researchers now have opportunities to develop more efficacious and targeted therapies. On the other hand, in breast cancer patients where AR acts as an oncogenic driver, in that situation the anti-androgenic therapy would be more beneficial. Indeed, a drug called bicalutamide has been studied and is currently undergoing a phase II clinical trial (www.clinicaltrials.gov; identifier NCT00468715). Similarly, another drug known as abiraterone that blocks androgen production by inhibiting the cytochrome p-450 (CYP17) is also being studied in a clinical trial (Table 3). Again, more extensive studies are required to identify which patients would be treated most appropriately by these therapies.

**Table 3:** List of clinical trials that are currently underway for assessing the efficacy and safety of the AR targets

Drugs (Trail ID)	Drugs details	Study population	Study phage (time)
Alpelisib and Enzalutamide (NCT03207529)	**Drug name (Alternative name)**: Alpelisib (BYL-719; NVP-BYL-719) **Type of drug**: Small Molecule **Mechanism(s) of Action**: Phosphatidylinositol 3 kinase alpha inhibitor **Generally used for**: Phase III Breast cancer; Phase II Non-small cell lung cancer; Phase I/II Colorectal cancer; Head and neck cancer; Multiple myeloma; Oesophageal cancer; Phase I Gastric cancer; Gastrointestinal stromal tumours; Pancreatic cancer; Rectal cancer; Uveal melanoma **Drug name (Alternative name)**: Enzalutamide (Xtandi) **Type of drug**: Small Molecule **Mechanism(s) of Action**: Competitive inhibitor of androgen receptor **Generally used for**: Treatment of castration-resistant prostate cancer. FDA approved on August 31, 2012	AR-positive and PTEN positive metastatic breast cancer patients	Phase 1(August 2017 to August 2019)
PalbociclibBicalutamide(NCT02605486)	**Drug name (Alternative name)**: Palbociclib (Ibrance) **Type of drug**: Small Molecule **Mechanism(s) of Action**: CDK4/6 inhibitor (DK4/6-cyclin D1 complex initiate's cell-cycle transition from G1 to S phase) **Generally used for**: Treatment of postmenopausal women with estrogen receptor (ER) positive, HER2 negative advanced breast cancer. **Drug name (Alternative name)**: Bicalutamide (Casodex) **Type of drug**: Small Molecule **Mechanism(s) of Action**: Competitive inhibitor of androgen receptor **Generally used for**: Treatment of men with advanced prostate cancer at stage D2, when there is evidence of metastases (cancer spread) to other areas of the body.	AR-positive metastatic breast cancer	Phase 2(November 2015- November 2018)
Taselisib and Enzalutamide(NCT02457910)	**Drug name (Alternative name)**: Taselisib **Type of drug**: Small Molecule **Mechanism(s) of Action**: PI3K inhibitor **Generally used for**: Treatment of HER2-negative, hormone receptor positive metastatic breast cancer patients.	AR-positive metastatic TNBC	Phase 2(June 2015 to December 2019)
Enzalutamide Trastuzumab (NCT02091960)	**Drug name (Alternative name)**: Trastuzumab (Herceptin) **Type of drug**: Monoclonal antibody **Mechanism(s) of Action**: Trastuzumab binds to the HER2 (or c-erbB2) proto-oncogene **Generally used for**: Treatment of HER2^+^ metastatic (spread) breast cancer.	AR-positive, HER2 amplified metastatic or locally advanced breast cancer	Phase 2(August 2014 to February 2017)
Enzalutamide and Taxol (NCT02689427)	**Drug name (Alternative name)**: Taxol (Paclitaxel) **Type of drug**: Small Molecule **Mechanism(s) of Action**: It binds to beta-tubulin subunits of microtubules and inhibits microtubule growth. **Generally used for**: Treatment of breast, ovarian, lung, bladder, prostate, melanoma, esophageal tumor	AR-positive TNBC, stage I–III breast cancer (neoadjuvant therapy)	Phase 2(September 2016 to September 2020)
Enzalutamide (NCT02750358)	**Drug name (Alternative name)**: Enzalutamide (Xtandi) **Type of drug**: Small Molecule **Mechanism(s) of Action**: Competitive inhibitor of androgen receptor **Generally used for**: Treatment of castration-resistant prostate cancer. FDA approved on August 31, 2012	AR-positive TNBC, stage I–III breast cancer (adjuvant therapy)	Phase 2(May 2016 to May 2019)
Abiraterone Acetate (NCT00755885)	**Drug name (Alternative name)**: Abiraterone Acetate(Zytiga) **Type of drug**: Small Molecule **Mechanism(s) of Action**: Inhibitor of the steroidal enzyme CYP17A1 **Generally used for**: Treatment of metastatic castration-resistant prostate cancer who have already received prior chemotherapy containing docetaxel.	Postmenopausal Women With Advanced or Metastatic Breast Cancer	Phase 2(October 2008 completed on June 2016)
AZD8186 (NCT01884285)	**Drug name (Alternative name)**: AZD8186 **Type of drug**: Small Molecule **Mechanism(s) of Action**: Phosphatidylinositol-4, 5-bisphosphate 3-kinase, catalytic subunit beta (PIK3CB; PI3Kb) and delta (PIK3CD; PI3Kd) inhibitor **Generally used for**: AZD8186 effectively inhibits growth of prostate and TNBC tumors	Advanced TNBC	Phase 1(July 2013 to September 2019)
Orteronel (NCT01990209)	**Drug name (Alternative name)**: Orteronel **Type of drug**: Small Molecule **Mechanism(s) of Action**: Orteronel (TAK-700) is a nonsteroidal CYP17A1 inhibitor **Generally used for**: Treatment of Prostate Cancer	AR-positive metastatic breast cancer	Phase 2(March 2014 to March 2018)
VT-464 (NCT02580448)	**Drug name (Alternative name)**: Seviteronel (VT-464) **Type of drug**: Small Molecule **Mechanism(s) of Action**: Nonsteroidal CYP17A1 inhibitor **Generally used for**: Treatment for prostate cancer	Advanced breast cancer. Phase I: TNBC or ER-positive, HER2 negativePhase II: AR-positive TNBC or ER-positive, HER2 negative	Phase 2(August 2015 to July 2017)
GTx-024 (NCT02368691)	**Drug name (Alternative name)**: Enobosarm (GTx-024) **Type of drug**: Small Molecule **Mechanism(s) of Action**: Non-steroidal selective androgen receptor modulator (SARM) **Generally used for**: Treatment of Stress Urinary Incontinence and Triple Negative Breast Cancer.	AR-positive advanced TNBC	Phase 2(June 2015 completed on December 2016)
Pembrolizumab and Enobosarm (NCT02971761)	**Drug name (Alternative name)**: Pembrolizumab **Type of drug**: Humanized monoclonal antibody **Mechanism(s) of Action**: It targets the programmed cell death 1 (PD-1) receptor **Generally used for**: Treatment of unresectable or metastatic melanoma **Drug name (Alternative name)**: Enobosarm (GTx-024) **Type of drug**: Small Molecule **Mechanism(s) of Action**: Non-steroidal selective androgen receptor modulator (SARM) **Generally used for**: Treatment of Stress Urinary Incontinence and Triple Negative Breast Cancer.	Androgen Receptor Positive Metastatic Triple Negative Breast Cancer patients	Phase 2(April 2017to June 2018)

## DISCUSSION

In this review, we discussed the complex AR signaling pathway in ER^+^ breast tumor under the condition of hormonal levels of post-menopausal women. We noticed a lot of variability of results *in vitro* study conditions, may be due to the limitation of availability of limited AR^+^ cell lines and their different sub-clones; use of the various androgens, their concentrations, and duration of treatments may also interfere with the findings [101]. Some labs also used cells from fresh biopsy material of early stage or metastatic in origin of breast tumors. Again co-expression of AR on different subtypes of cancers (ER, HER2 or in TNBC) are not well studied. Apart from these, various studies used different cut-off values for AR (of ≥1%, ≥5% or ≥10%) in IHC to determine AR positivity. Hence due to this lack of guidelines, it's difficult to compare the prognostic value of AR between different studies.

While reviewing studies, we observed that some subtypes of ER^+^ tumors showed that AR-agonist might be beneficial while other revealed that AR-antagonist has a positive effect on breast cancer. So, it's impotent to study further to determine which sub-types will be treated with which types of treatments. We also noticed there are inconsistency and need for the more relevant *in vivo* model system to study these effects. A previous *in vivo* study of AR function in metastasis of ER^+^ tumors used a breast cancer cell line xenograft and PDX models [21], which are very difficult to establish [39] and require non-physiological hormone supplements [37]. Therefore, we encourage researchers to test the AR-mediated metastatic growth of ER^+^ tumors in the mouse intraductal (MIND) model [38], which shows 30-100% engraftment rates and doesn't require non-physiological hormone supplements [38]. It is also possible that expression of progesterone receptor (PR) and human epithelial growth factor receptor 2 (HER2) can interfere the findings. Hence, we also encourage to use triple-negative breast cancer cell line (which doesn't express PR, ER, and HER2) to know effect only AR in breast cancer. As androgen and estrogen are inter-convertible, we also encourage checking endogenous E_2_ and DHT level in serum these mice. A previous study also showed that absence of E_2_ MCF-7 cells up-regulates aromatase, which can metabolize androgens to estrogens [33]. To solve this problem, we suggest checking aromatase (CYP19) expression (western blot analysis); if it's expressed at high levels, then they can treat the MCF-7 cells with AIs along with these hormone treatments.

The role of AR in breast cancer is evolving rapidly; a recent study showed that AR has a role in breast cancer development and metastasis [102]. In this review article, we discussed most of the relevant studies which have been published regarding the pathophysiology of AR on breast cancer mainly in post-menopausal women. We also focused on the crosstalk of AR and ER in tumorigenesis and metastasis in post-menopausal women and some of the therapeutic targets along the AR pathway. This review will bring insight to a therapeutic opportunity for ER^+^ breast cancer patients (~80% of patients) by AR-antagonist that may reduce metastasis. This study will also provide evidence to determine whether AR-targeted immunohistochemistry of breast cancer samples is a useful diagnostic tool for early detection of metastasis breast cancer. It is also important to study whether by blocking AR can prevent ER-regulated metastasis TamR ER^+^ breast cancer patients through a unique molecular mechanism other than anti-estrogen therapy.

## CONCLUSIONS

In this review, we discussed the effect of AR in breast cancer in post-menopausal women and the opportunities that lie ahead towards developing potential therapeutic strategies which either can be used as monotherapy or in combination with the existing treatment options. We also suggested that further studies in the area are urgently needed to use AR as a therapeutic target for post-menopausal women with breast cancers. Once the findings become available from the ongoing clinical trials with enobosarm and enzalutamide, we will have a better understanding of the pathway(s) that make post-menopausal women vulnerable to this dreaded oncogenic phenotype, and how to treat and diagnose them more effectively.

## Abbreviations

AR: Androgen Receptor
ER: Estrogen Receptor
Tam: Tamoxifen
DHT: Dihydrotestosterone
E_2_: 17β-estradiol
ERE: Estrogen Response Elements
PDX: Patient-Derived Xenograft
ChIP-Seq: Chromatin Immunoprecipitation Sequencing
MIND: Mouse Intraductal
HER2: Human Epidermal Growth Factor Receptor 2
TNBC: Triple-Negative Breast Cancer
